# SPRING: an RCT study of probiotics in the prevention of gestational diabetes mellitus in overweight and obese women

**DOI:** 10.1186/1471-2393-13-50

**Published:** 2013-02-25

**Authors:** Marloes Dekker Nitert, Helen L Barrett, Katie Foxcroft, Anne Tremellen, Shelley Wilkinson, Barbara Lingwood, Jacinta M Tobin, Chris McSweeney, Peter O’Rourke, H David McIntyre, Leonie K Callaway

**Affiliations:** 1 The UQ Centre for Clinical Research, The University of Queensland, RBWH campus, Butterfield street, Herston, QLD 4029, Australia; 2 School of Medicine, The University of Queensland, Herston, Australia; 3 The Royal Brisbane and Women’s Hospital, Herston, Australia; 4 Mater Medical Research Institute, Mothers’ & Babies Theme, South Brisbane, Australia; 5 Department of Nutrition & Dietetics, Mater Mothers’ Hospital, Brisbane, Australia; 6 Western Clinical School, The University of Melbourne, Melbourne, Australia; 7 CSIRO, St Lucia, Australia; 8 Statistics Unit, Queensland Institute of Medical Research, Herston, Australia; 9 Mater Clinical School, The University of Queensland, South Brisbane, Australia

## Abstract

**Background:**

Obesity is increasing in the child-bearing population as are the rates of gestational diabetes. Gestational diabetes is associated with higher rates of Cesarean Section for the mother and increased risks of macrosomia, higher body fat mass, respiratory distress and hypoglycemia for the infant. Prevention of gestational diabetes through life style intervention has proven to be difficult. A Finnish study showed that ingestion of specific probiotics altered the composition of the gut microbiome and thereby metabolism from early gestation and decreased rates of gestational diabetes in normal weight women. In SPRING (the Study of Probiotics IN the prevention of Gestational diabetes), the effectiveness of probiotics ingestion for the prevention of gestational diabetes will be assessed in overweight and obese women.

**Methods/design:**

SPRING is a multi-center, prospective, double-blind randomized controlled trial run at two tertiary maternity hospitals in Brisbane, Australia. Five hundred and forty (540) women with a BMI > 25.0 kg/m^2^ will be recruited over 2 years and receive either probiotics or placebo capsules from 16 weeks gestation until delivery. The probiotics capsules contain > 1x10^9^ cfu each of *Lactobacillus rhamnosus* GG and *Bifidobacterium lactis* BB-12 per capsule. The primary outcome is diagnosis of gestational diabetes at 28 weeks gestation. Secondary outcomes include rates of other pregnancy complications, gestational weight gain, mode of delivery, change in gut microbiome, preterm birth, macrosomia, and infant body composition. The trial has 80% power at a 5% 2-sided significance level to detect a >50% change in the rates of gestational diabetes in this high-risk group of pregnant women.

**Discussion:**

SPRING will show if probiotics can be used as an easily implementable method of preventing gestational diabetes in the high-risk group of overweight and obese pregnant women.

## Background

The incidence of gestational diabetes mellitus (GDM), —diabetes mellitus first diagnosed during pregnancy—, is on the rise in concordance with the rise in overweight and obesity in the obstetric population [1]. GDM is associated with short term and long term morbidity for mother and baby. In the short term, women with GDM have an increased risk of developing preeclampsia and delivery by Cesarean section [2,3]. For the infant, maternal GDM increases the risk of excessive adiposity, macrosomia (a birth weight of >4000 g), shoulder dystocia, admittance to the neonatal intensive care unit and neonatal hypoglycemia [4,5]. In the long term, maternal GDM is associated with increased risk of obesity as well as metabolic (type 2 diabetes) and cardiovascular disease in both mother and baby [6-8]. While current therapy, which is focused on normalizing blood glucose levels, is variably successful in reducing the short term complications of GDM, this may not be true for the longer term complications [9]. Therefore, prevention of GDM would be preferable.

The incidence of GDM is higher in women who are overweight or obese prior to falling pregnant [10]. Strategies for the prevention of GDM have until now been restricted to diet and exercise interventions, which have had mixed results [11,12]. Many of these trials have had small sample sizes and their methodological heterogeneity complicates comparability. Additionally, there are substantial barriers to pregnant women engaging in intensive lifestyle intervention programs [13,14].

The role of the gut microbiome as a modulator of metabolic and inflammatory process is currently being investigated. The gut microbiome is the composite of bacterial species present in the gut. Many factors influence the exact gut microbiome species composition in adult life including antibiotic therapy in early life [15], growth and inhibitory factors determined by diet, host and microbial factors as well as luminal pH, osmolarity and organism replication rates [16]. Gut microbiome species have discrete capabilities in generating energy for take-up by the host. The gut microbiome composition is altered in obese humans and animals, although weight loss causes changes in species composition to that resembling the gut microbiome of normal-weight individuals [17,18].

Ingestion of probiotics — “*microorganisms … able to confer defined health benefits on the host*” [19]— is an alternative pathway to alter gut microbiome species composition. The exact effects on metabolic and inflammatory processes in the host exerted by probiotics treatment are dependent on the genus and strain supplied in the probiotics. In the food chain, probiotic cultures are commonly present in variable amounts in dairy products such as yoghurts, fermented foods and some cheeses. It is not known to what extent these food sources alter the gut microbiome and thereby have biological effects outside research settings. Probiotic supplements are available from many outlets but effectiveness is dependent on especially temperature and anaerobic storage conditions, initial dose strain and quality. Probiotic supplementation in pregnancy is considered safe based on the limited safety data available [20].

A recent randomized controlled trial of probiotic intervention in normal weight women in pregnancy reported a decline in the rate of GDM from 34% to 13% with a combined dietary/probiotic supplementation approach [21]. To our knowledge, this trial has not been repeated and in particular, there has been no examination of whether probiotics might offer a useful therapeutic approach for overweight and obese women [22]. If probiotic supplementation were to offer substantial clinical benefit, it would provide a pragmatic approach to the prevention of GDM. This trial aims to provide an answer to these questions.

## Methods/design

### Study design

**Figure 1:**
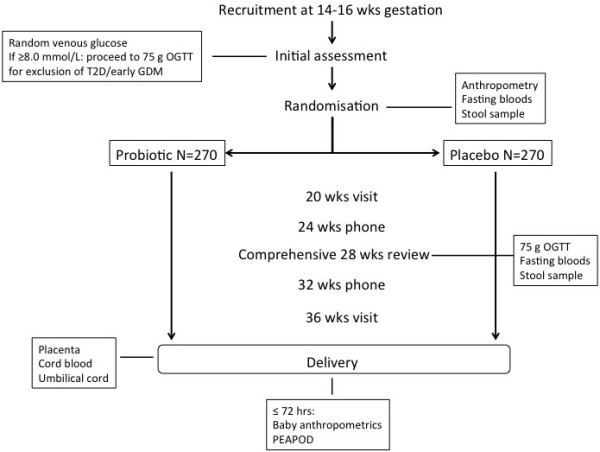
Recruitment at 14–16 wks gestation.

This is a prospective double blind randomized controlled trial, designed according to the CONSORT guidelines [23]. Our study will investigate the treatment of overweight and obese women with an oral probiotic combination using *Lactobacillus rhamnosus* GG and *Bifidobacterium animalis* subsp. *lactis* BB-12 (Chr. Hansen A/S, Horsholm, Denmark) at a dose of >1 x 10^9^ colony-forming units each per day or placebo (microcrystalline cellulose and dextrose anhydrate capsules – Chr. Hansen). Capsules will be taken once daily from enrolment prior to 16 weeks gestation until birth (Figure 1). 

### Outcomes

The primary outcome of the study is the frequency of diagnosis of GDM at 28 weeks gestation by a 75 g oral glucose tolerance test (OGTT) using the criteria of the International Association of the Diabetes and Pregnancy Study Group (IADPSG) [24].

A number of secondary outcomes will be assessed, which are listed in Table 1[25]. 

**Table 1:** Maternal, neonatal and process/intervention secondary outcomes assessed in SPRING

**Maternal**	**Neonatal**	**Process/intervention**
Gestational weight gain	Body composition, anthropometry	Visit attendance
Preeclampsia*	Preterm delivery	Adherence to probiotic/placebo regimen
Induction of labor	Shoulder dystocia	
Cesarean delivery	Hypoglycemia	
Elective	Treatment with supplementary fluids/feeds	
Emergency		
Change in prevalence of *L. rhamnosus* and *B. lactis* in gut microbiome	Nerve palsy	
Change in lipids and inflammatory profile	Admission to neonatal intensive care unit	
Change in dietary indices and physical activity levels between baseline and 28 weeks gestation	Jaundice requiring phototherapy	
	Bone fracture	
	Death: stillbirth or neonatal death	

* Preeclampsia as diagnosed as per the criteria of the international society for the study of hypertension in pregnancy (ISSHP) [25].

### Sample size justification/power calculation

At least one third of pregnant women cared for in Brisbane are overweight and obese, in line with the overweight and obesity rates for the general obstetric population in Australia [26]. In one year, across the Mater Mothers Hospital and the Royal Brisbane and Women’s Hospital, over 3300 overweight and obese pregnant women receive pregnancy care. We anticipate to recruit around 40 women per month across both centers with the recruitment strategy tested in a previous study [27]. Based on the Brisbane cohort within the Hyperglycemia and Adverse Pregnancy Outcome (HAPO) data [28], the rate of GDM amongst women who are overweight and obese is 19.3%. We expect that our screening strategy including a random venous plasma glucose and subsequent OGTT to confirm or exclude the diagnosis of overt diabetes, or early GDM will result in the exclusion of around 5% of women who were included in the HAPO study. This will reduce our expected prevalence of GDM to around 18% (5% of 19.3% is ~ 1%). The previous trial reported a 67% risk reduction [21]. However, even a risk reduction of 50% would be clinically meaningful, and we have based our power calculation on a 50% reduction. To detect a change in the incidence of GDM from 18% to 9%, with a power of 0.8, α of 0.05, and a two-tailed test, the estimated sample size is 226 per group. Allowing for 20% attrition of participants, we will recruit 270 women into each group. We anticipate that it will take 15 months to recruit this sample size.

### Inclusion criteria

The inclusion criteria for this study are: pregnant women at less than 16 weeks gestation; singleton pregnancy; body mass index (BMI) of >25.0 kg/m^2^; >18.0 years of age; able to read and understand English and provide informed consent.

### Exclusion criteria

The exclusion criteria for this study are: pregnancy at >16 weeks gestation at recruitment; multiple pregnancy; known pre-existing diabetes, impaired fasting glucose (IFG) or impaired glucose tolerance (IGT); GDM prior to recruitment as diagnosed by early pregnancy glucose testing described below; taking medications likely to influence glucose metabolism (in particular metformin, glucocorticoids and immunosuppressants); medical conditions associated with altered glucose metabolism (in particular Cushing’s syndrome/disease and hepatic cirrhosis); known major fetal abnormality noted on 12 week ultrasound examination; and known ingestion of probiotics via capsules or sachets.

The early pregnancy glucose testing follows a strategy supported by the guidelines of the IADPSG [29]. All women will have their random venous plasma glucose measured. Women with a random venous plasma glucose level of >11.0 mmol/L will be considered as having likely overt diabetes and will undergo appropriate further evaluation with fasting glucose, proceeding to an 75 g OGTT if required. Women with overt diabetes or early GDM will be excluded from this study.

In order to ensure participant safety, we do not want to enrol women with serious early pregnancy disturbances in glucose metabolism. Therefore, to err on the side of caution, we will refer any woman with a random venous plasma glucose between 8.0 and 11.0 mmol/L for a 75 g OGTT. Women who are diagnosed with early GDM on this OGTT will be referred for treatment, and excluded from this study. Conversely, those women with an initial elevated random venous plasma glucose but normal OGTT results will remain eligible for randomization.

### Recruitment facility

This study will be conducted at the Royal Brisbane and Women’s Hospital and the Mater Mothers Hospital in Brisbane, Australia. Both hospitals are major tertiary referral obstetric hospitals. Combined, they care for 10,000 public and 5,000 private births annually.

### Enrollment process

Our recruitment strategy is based on the successful strategy used in a previous pilot study [27]. All women enrolling for pregnancy care at the two centers will be sent a letter inviting them to participate in this study. Those who respond will be screened for inclusion and exclusion criteria during an initial phone call, and if eligible, will be invited to attend a baseline assessment.

### Baseline assessment

**Figure 2:**
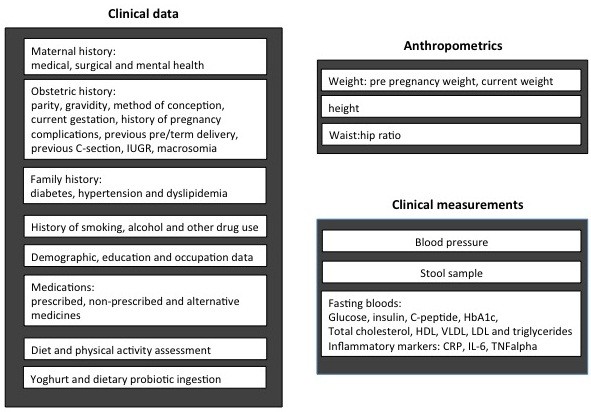
Baseline assessment at 16 weeks gestation including clinical history of the mother as well as anthropometric and clinical measurements.

At the baseline assessment at <16 weeks gestation after review of the inclusion/exclusion criteria and the initial random venous plasma glucose test, data will be obtained on clinical history and anthropometrics and clinical measurements will be taken (Figure 2).

Dietary intake will be assessed using the Cancer Council Victoria’s Dietary Questionnaire for Epidemiological Studies (Version 2) [30]. The validated food frequency questionnaire (FFQ) [31] comprises a food list of 74 items with 10 frequency response options ranging from ‘Never’ to ‘3 or more times per day’. The FFQ also contains three photographs of scaled portions for four foods (used to calculate a portion size calibrator), questions on the overall frequency of fruit and vegetable consumption, and questions on consumption of foods that do not fit easily into the frequency format (such as breads). The 74 food items are grouped into 4 categories: cereal foods, sweets and snacks; dairy products; meats and fish; fruit, and vegetables. A separate set of questions covers intake of alcoholic beverages. The food composition data used to calculate nutrients are from NUTTAB95, supplemented by other data where necessary. The output includes: kilojoules, detailed macronutrient and micronutrient profiles, as well as glycemic index, and glycemic load. The DQESv2 analysis will be performed by the Cancer Council Victoria following the instructions in the User Information Guide.

Self-reported physical activity will be assessed using the Active Australia Survey (AAS) [32]. The AAS assesses the frequency and duration of participation in walking, moderate, and vigorous physical activity in the past week. Physical activity data will be summed to determine total minutes of weekly physical activity, as per the AAS manual [32]. This captured time spent in activities of walking, vigorous exercise, and vigorous yardwork/gardening. It does not record household chores, occupational or incidental activity [32]. In calculation of minutes of physical activity, minutes of vigorous activity will not be weighted by two as suggested in the AAS manual [32], as we are interested in absolute minutes active, rather than the association between physical activity dose and health benefits that may be dependent on total energy expenditure. This allows the creation of the parametric variable “minutes of physical activity”. This will be capped at 840 minutes, according to the AAS manual [32].

Weight will be measured to the nearest 0.1 kg using a spring balance scale. Height will be measured with a wall mounted stadiometer to the nearest 0.5cm. Waist measurements will be taken with a flexible, inelastic tape measure with measures taken on the skin where the circumference is the smallest with the measure positioned horizontally, parallel to the floor with the patient standing up straight at the end of normal expiration. Hip measurements will be taken at the point where the hip circumference is greatest.

### Intervention

After the baseline assessment, women will be randomly allocated to probiotic or matching placebo trial capsules. Participants will be advised to take their capsule with a cold drink, excluding carbonated and sugared beverages, to maximize survival and potential benefit of the probiotics. All women will be provided with dietary advice that follows current Australian nutrition guidelines for pregnancy [33], including simple advice regarding recommended weight gain in pregnancy [34]. Further dietary advice will be provided as clinically indicated as part of standard clinical care.

### Randomization procedure

Participant randomization will occur based on computer-generated random number codes sealed in opaque envelopes. Participants will be stratified by center and by BMI category (BMI >25-30; BMI >30-40; BMI>40 kg/m^2^).

### Allocation concealment and blinding

Placebo and Probiotic supplements will be identically packaged and refrigerated. All medical staff, research assistants, nursing staff and participants will be masked to the randomized allocation.

### Clinical assessments

#### Comprehensive assessment at 28 weeks gestation

At 28 weeks gestation, all baseline clinical assessments, blood tests, stool samples, questionnaires and an updated medical history will be repeated. At this time point, a 75 g OGTT will be performed to identify women who have developed GDM (primary endpoint of the study).

#### Assessment at 36 weeks gestation

At 36 weeks gestation, weight, waist to hip ratio, blood pressure and medication compliance will be assessed by interview.

#### Post delivery assessment

At delivery, cord blood will be sampled, and umbilical cord and placenta will be collected in a subset of women. Neonatal anthropometry will be collected and fetal body composition will be measured by air displacement plethysmography in a PEAPOD Infant Body Composition System (Cosmed, Rome, Italy) [35] within 3 days after birth.

### Compliance

Compliance with probiotic ingestion will be assessed by: (1) participant interview; (2) monthly telephone follow-up; and (3) change in fecal microbiome at 28 weeks gestation.

### Retention optimization

All women independent of treatment allocation will be seen on at baseline, 28 and 36 weeks gestation, but will also receive a telephone consult monthly or more frequently as required. This is for monitoring of weight, blood pressure, pill count and early detection of adverse events. Women will also be offered further supply of capsules when needed. The additional monthly phone review is also expected to assist with adherence and retention.

### Confirmation of probiotic colonization

Maternal stool carriage of probiotic species will be documented to confirm colonization and quantify the number of *L. rhamnosus* and *B. lactis* species using stool samples. This will be done using a stool sample prior to the first dose of probiotic or placebo and repeated at 28 weeks gestation. DNA will be extracted from the stool sample using previously developed techniques [36,37]. Briefly, microbial DNA extraction involves an enzymatic lysis step followed by bead beating in the presence of high concentrations of sodium dodecyl sulfate salt and EDTA, with final DNA purification by a commercial clean-up kit (Qiagen). Extracted DNA will be tested by quantitative real-time PCR for *Lactobacillus rhamnosus* GG and *Bifidobacterium lactis* BB12 using strain specific primers and probes [38].

### Ethical considerations

The study has been approved by the human research ethics committees of the Royal Brisbane and Women’s Hospital, Mater Health Services and The University of Queensland. Any adverse events will be reported to the clinical trial management committee and the human research ethics committees involved.

### Trial Governance

An independent data monitoring committee, consisting of the Director of Pharmacy at the Royal Brisbane and Women’s Hospital, an ethicist and an independent clinician with clinical trials experience is appointed to assess the progress of the trial after completion of the data for each 100 participants enrolled. Any adverse events that might be due to the probiotics will be reported to the clinical trial management committee and the human research ethics committees of the Royal Brisbane and Women’s Hospital and Mater Health Services.

### Analysis

#### Analysis methods

Data will be analysed using the intention-to- treat principle and no intermediate analyses are scheduled. The primary outcome of the study is diagnosis of GDM at 28 weeks gestation. This will be assessed by a 75 g OGTT. The frequency of GDM will be compared between the groups with binary logistic regression accounting for the initial stratification by site and BMI catrgory. The secondary outcomes will be analyzed with general linear models for continuous outcomes and logistic regression for binary outcomes. All data will be summarized by mean and standard deviation for continuous variables and frequencies for categories.

## Discussion

### Anticipated limitations

In this randomized control trial, the capacity of two specific probiotic substrains to prevent GDM will be tested. A number of limitations can be envisioned: First, the number of organisms in the probiotic capsules given may be too low to prevent GDM. The dosage of the probiotics is based on the previous study performed in normal weight women [21], however overweight and obese women may require a higher dose. Second, the effect size of the probiotics may be lower than expected. Our power calculations are based on a reduction of the incidence of GDM by 50%. A 30% reduction would not reach statistical significance but would be clinically important. Third, there is a risk that women in both the probiotics and placebo group will perceive a potential advantage from probiotic ingestion and increase the intake of probiotics-containing foods such as yoghurts, even while they are requested not to, thereby blurring the results. The dietary questionnaire obtained at 28 weeks gestation is designed to identify this, should it occur. In all RCTs, there is the risk of non-compliance with the treatment. Regular encouraging mobile phone text messages, phone interviews and review visits will be conducted to improve compliance. At the review visits, capsule counting will be performed to assess compliance. Additionally, comparison of the stool samples provided at baseline and at 28 weeks gestation for the species provided by the probiotics will give an objective biological measure of compliance [39].

### Clinical significance of the study

This trial will evaluate the efficacy of probiotic ingestion from early pregnancy to prevent GDM in a high-risk group of women who are overweight and obese. If successful, probiotics would offer a therapeutic option for the prevention of GDM that is simple, cheap and easy to incorporate into standard clinical care.

### Trial status

This RCT started enrolling in November 2012. Currently eight participants have been enrolled.

## Competing interests

The probiotics and placebo capsules have been donated by Chr. Hansen A/S. None of the authors has any competing interests.

## Authors’ contributions

MDN, HLB, HDM and LKC conceived and designed the study. MDN wrote the manuscript. HLB, HDM and LKC helped draft the manuscript. SW advised on diet, physical activity, and anthropometric assessment, measurement, and analysis. KF, BL, JMT, CM assisted with study design and manuscript revision. POR contributed to study design, sample size calculations and the statistical analysis plan. All authors read and approved the final manuscript.

## Pre-publication history

The pre-publication history for this paper can be accessed here:

http://www.biomedcentral.com/1471-2393/13/50/prepub
